# Self-regulation learning as active inference: dynamic causal modeling of an fMRI neurofeedback task

**DOI:** 10.3389/fnins.2023.1212549

**Published:** 2023-08-15

**Authors:** Gabriela Vargas, David Araya, Pradyumna Sepulveda, Maria Rodriguez-Fernandez, Karl J. Friston, Ranganatha Sitaram, Wael El-Deredy

**Affiliations:** ^1^Institute for Biological and Medical Engineering, Schools of Engineering, Medicine and Biological Sciences, Pontificia Universidad Catolica de Chile, Santiago, Chile; ^2^Brain Dynamics Lab, Universidad de Valparaíso, Valparaiso, Chile; ^3^Instituto de Tecnología para la Innovación en Salud y Bienestar, Facultad de Ingeniería, Universidad Andrés Bello, Viña del Mar, Chile; ^4^Institute of Cognitive Neuroscience, University College London, London, United Kingdom; ^5^Department of Psychiatry, Columbia University, New York, NY, United States; ^6^Wellcome Centre for Human Neuroimaging, Institute of Neurology, University College London, London, United Kingdom; ^7^St. Jude Children's Research Hospital, Memphis, TN, United States; ^8^Valencian Graduate School and Research Network of Artificial Intelligence, Valencia, Spain; ^9^Department of Electronic Engineering, School of Engineering, Universitat de València, Valencia, Spain

**Keywords:** neurofeedback, brain-computer interface, fMRI, Active Inference, self-regulation learning

## Abstract

**Introduction:**

Learning to self-regulate brain activity by neurofeedback has been shown to lead to changes in the brain and behavior, with beneficial clinical and non-clinical outcomes. Neurofeedback uses a brain-computer interface to guide participants to change some feature of their brain activity. However, the neural mechanism of self-regulation learning remains unclear, with only 50% of the participants succeeding in achieving it. To bridge this knowledge gap, our study delves into the neural mechanisms of self-regulation learning via neurofeedback and investigates the brain processes associated with successful brain self-regulation.

**Methods:**

We study the neural underpinnings of self-regulation learning by employing dynamical causal modeling (DCM) in conjunction with real-time functional MRI data. The study involved a cohort of 18 participants undergoing neurofeedback training targeting the supplementary motor area. A critical focus was the comparison between top-down hierarchical connectivity models proposed by Active Inference and alternative bottom-up connectivity models like reinforcement learning.

**Results:**

Our analysis revealed a crucial distinction in brain connectivity patterns between successful and non-successful learners. Particularly, successful learners evinced a significantly stronger top-down effective connectivity towards the target area implicated in self-regulation. This heightened top-down network engagement closely resembles the patterns observed in goal-oriented and cognitive control studies, shedding light on the intricate cognitive processes intertwined with self-regulation learning.

**Discussion:**

The findings from our investigation underscore the significance of cognitive mechanisms in the process of self-regulation learning through neurofeedback. The observed stronger top-down effective connectivity in successful learners indicates the involvement of hierarchical cognitive control, which aligns with the tenets of Active Inference. This study contributes to a deeper understanding of the neural dynamics behind successful self-regulation learning and provides insights into the potential cognitive architecture underpinning this process.

## 1. Introduction: self-regulation learning via neurofeedback

**Figure 1:**
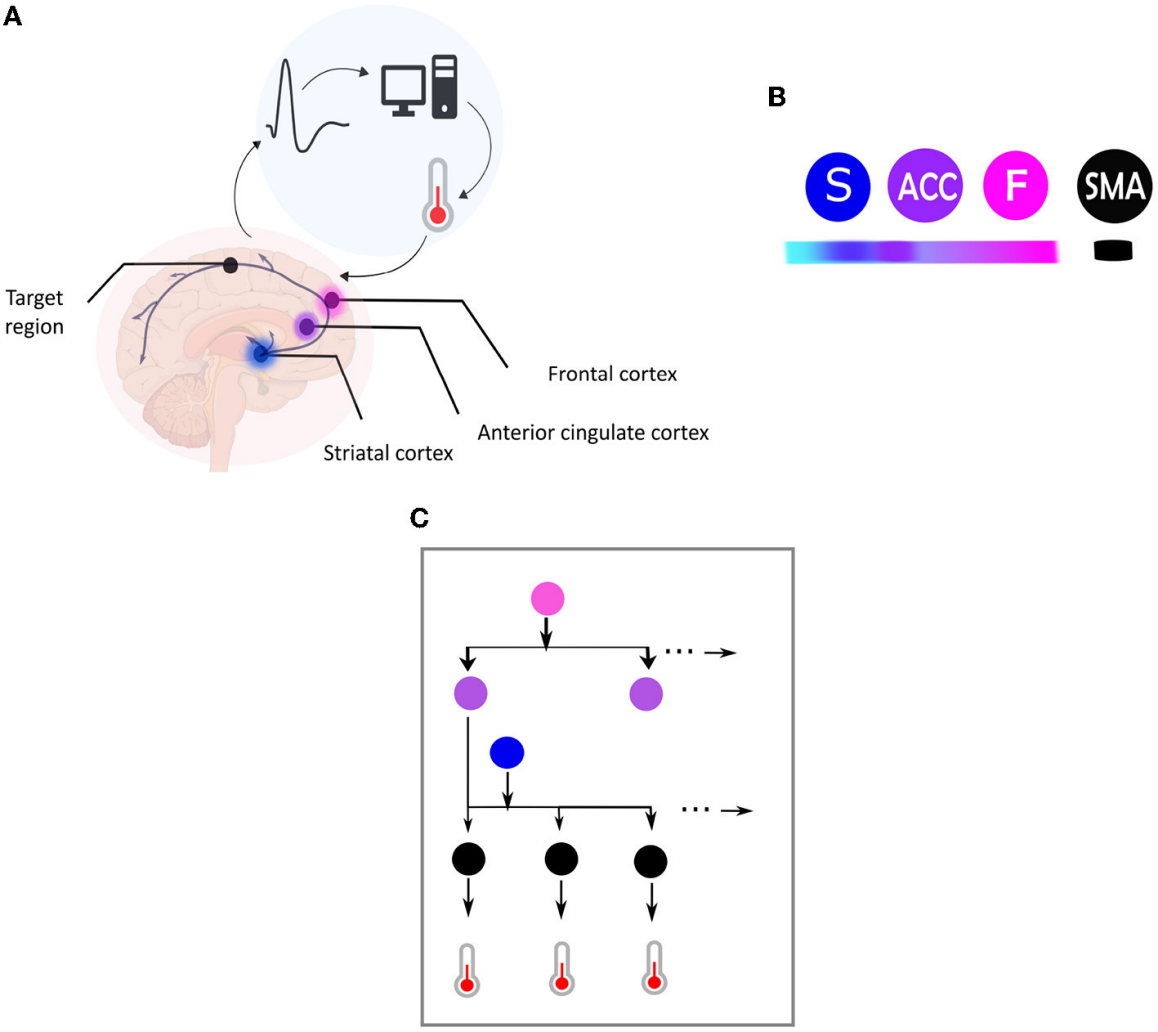
**(A)** Central elements in a neurofeedback system. This figure illustrates the main components of a neurofeedback system, which involves capturing brain signals from a target area (indicated by the black dot) and converting them in real-time into sensory feedback (e.g., visual feedback using a thermometer), representing the feature of the brain activity to be regulated (e.g., amplitude of the BOLD response). The participant then uses the sensory signal to regulate their brain activity in the target area. The color code used is followed for the different elements depicted in **(B)**. **(B)** This figure illustrates the hierarchical organization of brain areas based on the Active Inference framework, using color coding to map brain nodes. The ventromedial prefrontal cortex is labeled as “F” in fuchsia pink, the striatum as “S” in blue, and the anterior cingulate cortex as “ACC” in purple. Higher levels of the hierarchy are highlighted in darker fuchsia, while lower levels are highlighted in lighter blue. The black node represents the target region, which is the supplementary motor area (SMA). **(C)** Brain self-regulation from the perspective of Active Inference. This figure illustrates the brain self-regulatory network as a directed graph based on the computational anatomy of Active Inference. The nodes in the network correspond to brain regions, and are color-coded according to their hierarchical level: ventromedial prefrontal cortex (F) in fuchsia pink, anterior cingulate cortex (ACC) in purple, striatum (S) in blue, and the target region (SMA) in black **(B)**. The edges in the graph denote directed connections between brain regions. The hierarchical structure of the graph allows for inference and learning of how to control the activity of the target area through sensory input from the thermometer. To control thermometer movement, it is necessary to have low-level control of the striatum, mid-level control of the ACC, and high-level control of the F node. This self-regulation network is adapted from Emmert et al. (2016) and the Active inference anatomy based on Pezzulo et al. (2015).

Neurofeedback (NF) is a technique within the field of brain-computer interfaces (BCI) that involves providing individuals with real-time information about their neural processes. Activity-based NF aims to enable individuals to change and control their brain activity (Sitaram et al., 2017). By self-regulation, subjects can learn to exert control over their neural patterns, leading to substantial clinical benefits for a wide range of neurological and psychiatric disorders (Amano et al., 2016; Emmert et al., 2016; Watanabe et al., 2017; Yamashita et al., 2017; Haugg et al., 2020). NF has been shown to modify brain and behavioral functions by selectively targeting brain areas and by repeatedly training with contingent feedback and reward (Ramot and Martin, 2022). The technique involves presenting individuals with real-time feedback of their own brain activity, allowing them to learn how to regulate their brain function by associating changes in their brain activity with specific self-regulatory actions, targeting a broad range of brain regions (Thibault et al., 2018; Haugg et al., 2020) (Figure 1A). However, the underlying brain mechanisms that support self-regulation learning by NF are not yet fully understood. Recent advances in neuroimaging and computational neuroscience provide a unique opportunity to investigate the underlying neural mechanisms of self-regulation learning by NF. Research progress has shed light on the networks involved in brain regulation learning (Emmert et al., 2016). Real-time functional MRI (rt-fMRI) has shown that, along with changes in the target area, changes in brain dynamics during self-regulatory training have also been reported (Watanabe et al., 2017). These dynamic changes are closely linked to associative reinforcement learning mechanisms (Sitaram et al., 2017; Watanabe et al., 2017; Shibata et al., 2019; Skottnik et al., 2019; Cortese et al., 2020). Neuroimaging studies have revealed neural activity within the cortico-cortical and cortico-basal loops, involving key areas such as the striatum, anterior cingulate cortex (ACC), orbitofrontal cortex (OFC), anterior insula cortex (AIC), and dorsolateral prefrontal cortex (dlPFC). These neural correlates are consistent with activity patterns observed in reinforcement learning (Rao and Ballard, 1999; Mnih et al., 2015; Huang et al., 2020). Previous studies have investigated reinforcement factors in NF training protocols. For instance, studies have shown that protocols incorporating contingent feedback and implicit instructions tend to result in more effective automatic learning compared to protocols utilizing intermittent feedback (Oblak et al., 2017). This observed effect may be attributed to the time required for updating and processing the reward signal during the automatic learning process. However, it is important to acknowledge that there are varying findings regarding the effectiveness of feedback types. While some studies suggest that intermittent feedback leads to superior outcomes, others find no significant effect on the success of NF interventions. The variability in these findings can be attributed to the complexity of the self-regulation mechanism involved in NF training, which encompasses not only automatic learning processes but also the utilization of cognitive strategies. Overall, NF shows a wide range of inter-subject variability during training (Thibault et al., 2018; Haugg et al., 2020). Indeed, little is known about the mechanisms of learning to self-regulate or why learning rates are relatively low. It is worth noting the existence of a phenomenon known as the “non-responder effect,” where a significant portion of participants (as low as 25% in some studies) are unable to effectively learn the task in the context of real-time functional MRI (rt-fMRI) neurofeedback (Fede et al., 2020). It remains a challenge to understand brain-based mechanisms that underlie learning self-regulation and its associated aspects, such as sustainability and transferability (Sitaram et al., 2017; Alkoby et al., 2018; Thibault et al., 2018; Shibata et al., 2019; Skottnik et al., 2019; Fede et al., 2020; Haugg et al., 2020). In this context, we propose to employ the normative framework of Active inference and the free energy principle (FEP) as a general account of sentient behavior (Moran et al., 2014; Friston et al., 2016, 2017; Pezzulo et al., 2018; Chen et al., 2020). Active inference formalizes the brain as a statistical organ that makes inferences about the environmental causes of its sensations. This approach offers a theoretical framework to explain how the brain learns to self-regulate its activity by minimizing prediction errors and optimizing its internal representations of the external environment (Friston et al., 2015, 2017). Both simulations and neurophysiological data have demonstrated the model's predictive power (Moran et al., 2014; Friston et al., 2017; Parr et al., 2019; Da Costa et al., 2020). Active inference thus provides a process theory for various brain phenomena, including neural dynamics, such as dopamine responses, and cognitive processes, such as learning. Most relevantly, it includes mechanistic insights into the brain and behavior, predicted in biophysical message-passing schemes (Friston et al., 2015, 2016, 2017; Pezzulo et al., 2018; Chen et al., 2020; Da Costa et al., 2020). Active inference proposes a hierarchical computational anatomy as the neural substrate that scaffolds the internal dynamics of the brain (Pezzulo et al., 2015, 2018; Friston et al., 2017; Chen et al., 2020; Da Costa et al., 2020; Smith et al., 2020) (Figure 1B). For example, we would expect NF to engage hierarchical brain networks with top-down connectivity; in which case learners will exhibit stronger connections between the frontal and target area. Figure 1C illustrates the main elements in an NF experiment setup from the Active inference perspective. To investigate NF's neural mechanisms of self-regulation learning, we use dynamic causal models (DCM) (Friston et al., 2003; Daunizeau et al., 2011; Zeidman et al., 2019) to compare effective connectivity between brain regions for two principal network structures: top-down Active inference and bottom-up reinforcement learning (RL) (Pezzulo et al., 2018; Huang et al., 2020). We hypothesized that an Active inference network model is more consistent with neurofeedback training than a RL model based on the reward system and bottom-up connections. We use model comparison (Stephan et al., 2009; Penny et al., 2010) to assess which network structure is more likely to have generated the observed fMRI data from a previously published NF study. We then test the differences between participants with successful modulation of brain activity that increased from the beginning to the end of the intervention (learners) and non-learners of the NF self-regulation task for the most probable network. We chose this dataset (Sepulveda et al., 2016) because it aimed to compare the drivers of brain self-regulation learning and explore neural substrates of brain hemodynamic control. We summarize the experiment in Section 2.

## 2. Materials and methods

Details about the data acquisition, the participants, and the neurofeedback training can be found in Sepulveda et al. (2016). For completeness, the participant characteristics and neurofeedback training are repeated here.

### 2.1. Participants

Twenty naive human male volunteers, right-handed, aged 18–35 years, without any history of previous psychiatric or neurological disorders, took part in the study. Before the experiment, participants were instructed to regulate their supplementary motor area (SMA) activity with the help of visual neurofeedback. The instruction includes an explanation of the neurofeedback thermometer display and the objective to up-regulate by increasing the bars of the thermometer. Participants were randomly distributed in four groups of equal size (*n* = 5), matched by age. The following were the four groups of participants: Group “GF”, the participants of this group received only contingent feedback (F) from SMA. Group “GF,I”, the participants of this group received contingent feedback (F) from SMA and were instructed that feedback was proportional to the activity of a movement-related area of the brain; hence were encouraged to use motor imagery. Group “GF,R”: Participants were given contingent feedback and monetary reward (R) proportional to the increase in the BOLD signal in the SMA at the end of each up-regulation block. Group “GF,I,R”: Participants were given contingent feedback, monetary reward and instructions for motor imagery. Out of the initial cohort of twenty recruited participants, two subjects from the GF,I group were excluded from our analysis due to incomplete data.

### 2.2. Neurofeedback training

Participants took part in a 2-day neurofeedback training sessions. Previous to each session, SMA (ROI) was delineated using both a functional localizer and anatomical references. The protocol included four training runs and one transfer run in each training session. Each training run alternated between 4 baselines (rest) and 3 up-regulation blocks in a block design. The transfer session was included at the end of the training runs to assess the changes due to NF as a post-training test. Transfer sessions consisted of self-regulation run without feedback. During the baseline blocks, the target level indicator of the thermometer display remained static, which indicated to the participants that they should remain at rest. During the up-regulation blocks, contingent feedback was provided. The bars level indicator showed increment or decrement in correspondence with the feedback calculation, which indicated to the participants BOLD signal changes compared to the immediately preceding baseline block. Groups with monetary rewards have visually presented the value of their monetary reward in the last 3 s (2 volumes) of the block, using an image indicating the amount of money earned corresponding to the increase in the BOLD signal in the previous up-regulation block.

### 2.3. Dynamic causal modeling

**Figure 2:**
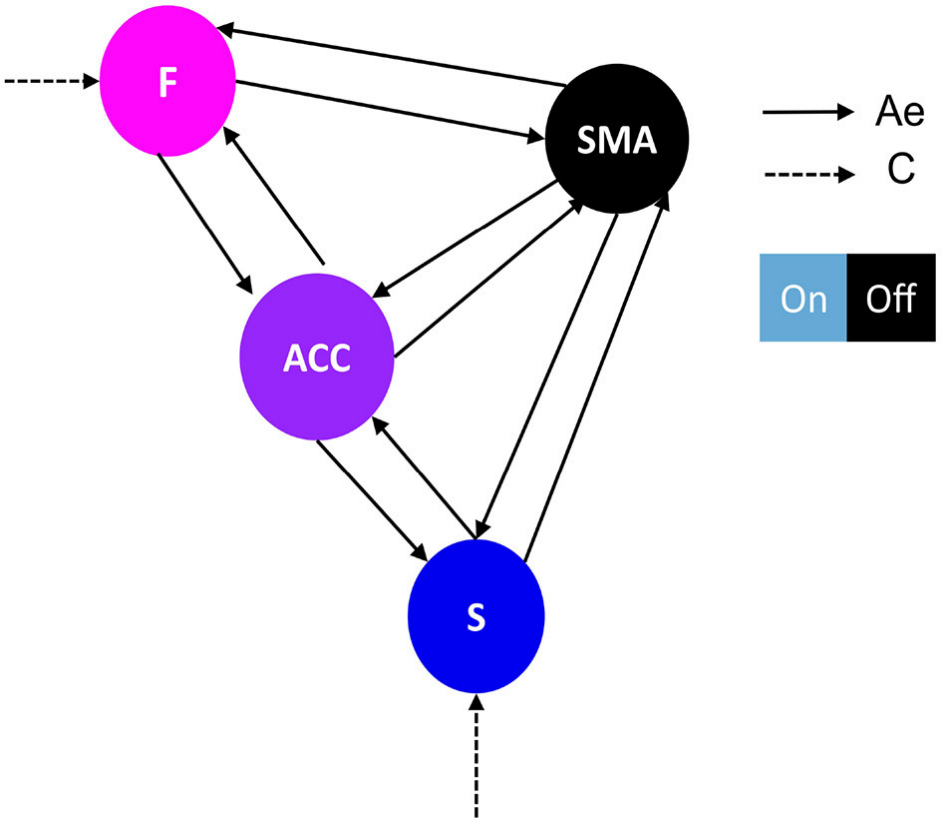
Network architecture used for the first family-level inference. This figure shows the base schema upon which we tested 14 network models. Correlation matrices in Figures 4A, B illustrate variations in effective connectivity (Ae matrix) and conduction input (C matrix) and indicate which parameters were estimated from the data. The network follows a simplified Ae architecture proposed by Emmert et al. (2016), characterized by the F-ACC-S axis, which is color-coded based on their hierarchical level according to Pezzulo et al. (2015). The type of driving input (matrix C) determines the family type, either frontal family (F) (Figure 4A) or striatal family (S) (Figure 4B). The nodes of the network include: F, frontal: Superior and middle frontal gyrus, AAL3 numbers 3,4,5,6; SMA, supplementary motor area: right supplementary motor area, AAL3 number 16; ACC, anterior cingulate cortex: subgenual, pregenual, and supracallosal parts, AAL3 numbers 151, 152, 153, 154, 155, 156; S, striatum: Putamen and nucleus accumbens, AAL3 numbers 78, 80, 157, 158. The effective connectivity is represented by a continuous black arrow, and the input is represented by a dotted black arrow. The DCM matrix representation is based on Zeidman et al. (2019).

**Figure 3:**
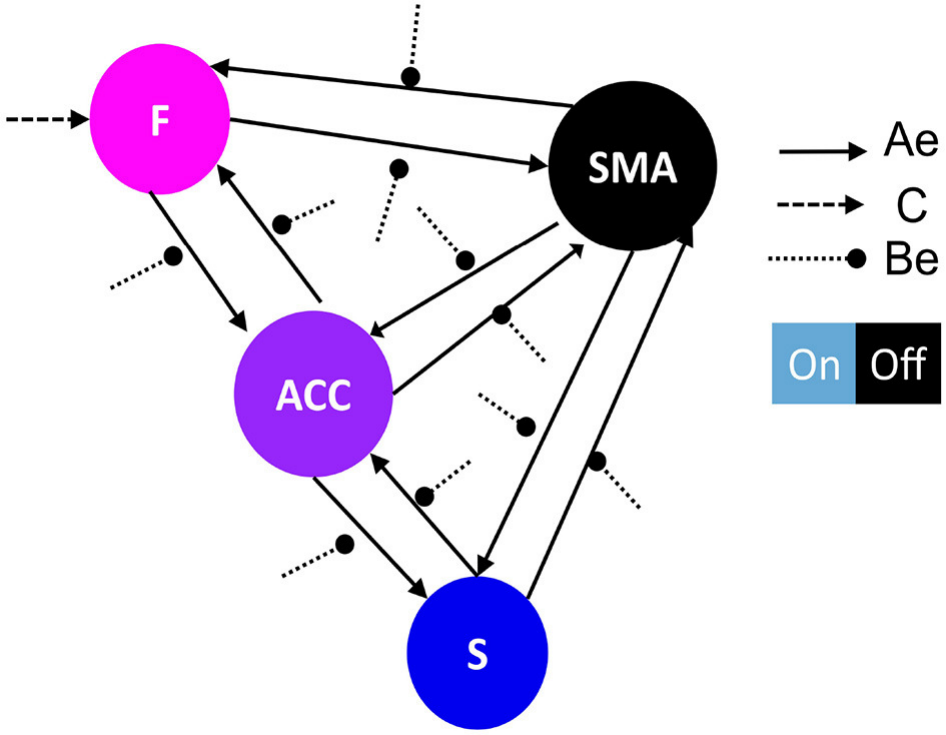
Network architecture used for the second family-level inference. This figure illustrates the base schema on which we tested 13 network models to compare possible effective connectivity architecture. Variations in the modulation of extrinsic connectivity (matrix Be) are shown as correlation matrices in Figures 5A–C, indicating which parameters were estimated from the data. Depending on the source node that the modulation points to, we determine the family type; F frontal family (matrix definition on Figure 5B), ACC family (matrix definition on Figure 5A), and S Striatal family (matrix definition on Figure 5C). The modulatory connectivity is indicated by the black dot arrow. Color-coded based on their hierarchical level according to Pezzulo et al. (2015). DCM matrix representation based on Zeidman et al. (2019).

For our analysis, we used SPM 12.5 version as implemented in SPM 12 in Matlab. We employed the one-state bilinear DCM as our neuronal model due to its simplicity which enables efficient inference about (changes in) coupling parameters (Friston et al., 2003; Daunizeau et al., 2011). Based on the experimental design and first-level analysis conducted in Sepulveda et al. (2016), the visual feedback information was modeled as the driving input (matrix C) as it was the onset of all runs. In addition, the up-regulation effect was modeled as modulation of effective connectivity in the runs, due to that alters the connection between regions (Daunizeau et al., 2011; Friston et al., 2019; Zeidman et al., 2019). We used Variational Laplace to invert and estimate the model evidence and connectivity parameters of each model given fMRI data for each subject and run by concatenating all eight runs. Due to the inter-participant variability in our study and in order to generalize the results to the population, we used a random effect (RFX) Bayesian model selection (BMS) approach for our DCM analysis (Stephan et al., 2009; Penny et al., 2010; Friston et al., 2016; Chen et al., 2020). BMS-RFX involves studying inter-individual differences with multiple models to differentiate between subjects, which allows group-level model inference. Model evidence is approximated with an evidence lower bound, a.k.a. variational Free Energy (Friston et al., 2007; Daunizeau et al., 2011). Using Bayesian model selection (BMS), information over models in each model family was pooled and compared collectively. Results were reported as protected exceedance family probabilities index (pxp) separately for each experimental group. A probability higher than 0.80 indicated the dominance of one particular model family compared to the other model families (Stephan et al., 2009; Penny et al., 2010). We employed a two-step hierarchical approach to identify the general model structure that underlies successful brain self-regulation. In the first step, we conducted family-level inference procedures to investigate the optimal baseline effective connectivity architecture (matrix A), particularly the between-region connections, which are defined as the “Ae” matrix, and the region that received the driving input of feedback (matrix C) (Figure 2 for a graphical description). We held all regions with self-inhibitory within-region connections fixed during the analysis. In the second step, we conducted family-level inference procedures to investigate the optimal modulation of effective connectivity by experimental conditions architecture (matrix B), specifically the between-region connections, defined as the “Be” matrix (Figure 3). Subsequently, we used parameter-level inference procedures to investigate which changes in connectivity strength mediated learning to up-regulate the SMA. The analysis was carried out for the two groups, i.e., the learners (*N* = 9) and the non-learners (*N* = 9).

We selected the four regions for analysis based on consistent activation observed in all study groups and trials in the Sepulveda et al. (2016) study. These regions included the bilateral supplementary motor area (SMA) and superior frontal gyrus (SFG) as the frontal lobe (F), the anterior cingulate cortex (ACC) comprising subgenual, pregenual, and supracallosal parts, and the striatum (S). To extract the BOLD time series from these regions, we used the principal eigenvariate within an anatomical mask of each area as defined by the Automated Anatomical Labeling 3 (AAL3) (Rolls et al., 2020). The up-regulation and baseline contrast maps obtained from the fMRI study were also used to identify activity in these regions.

#### 2.3.1. Model specification: family-level inference

**Figure 4:**
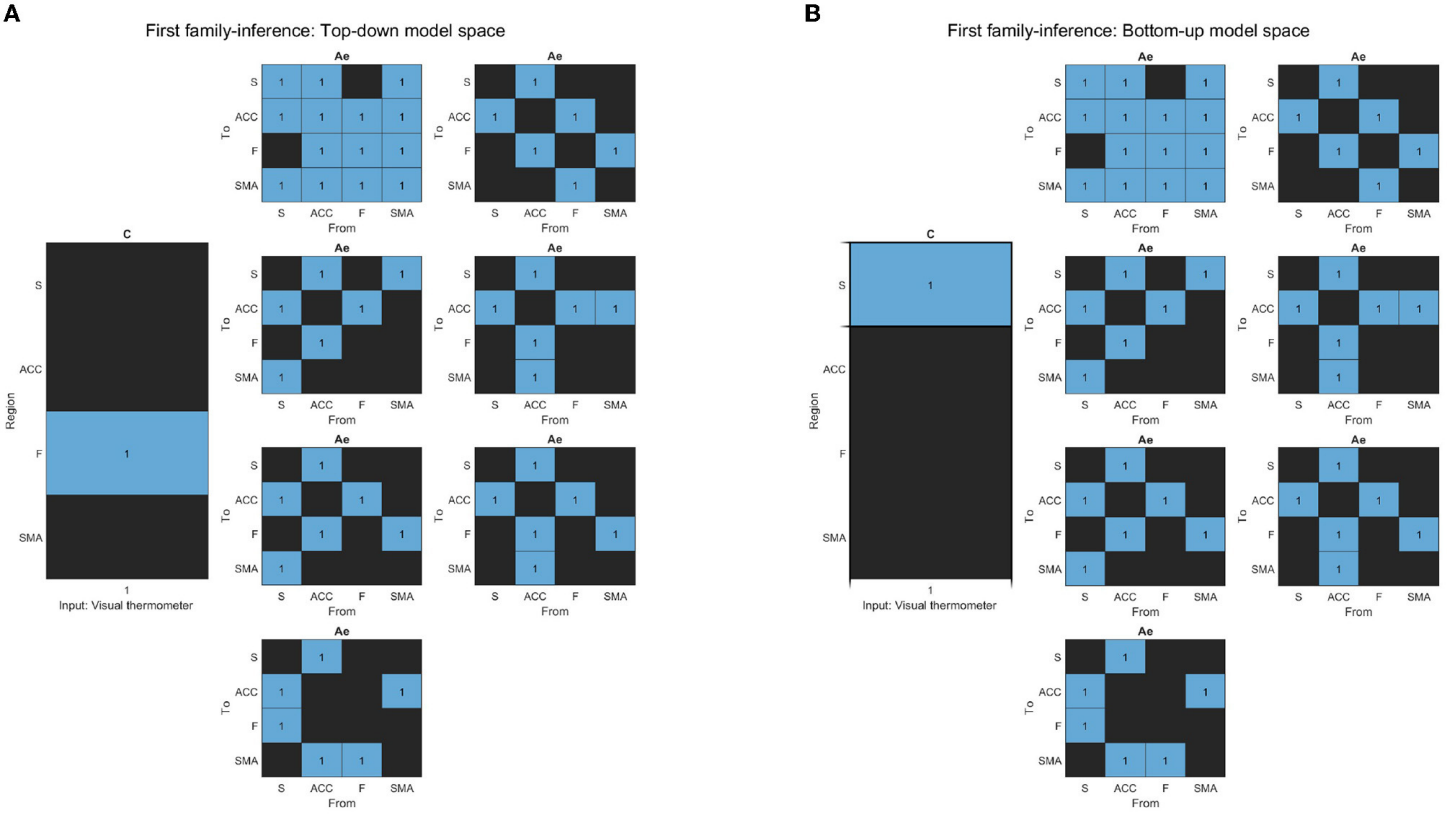
**(A)** The figure displays the correlation matrices corresponding to the “Top-down” sub-set of models from the first family-level inference. The left side of the figure shows the matrix C driving input, with the “Frontal node” as the parameter to be estimated, indicated in light blue, while the rest of the nodes are fixed at zero (switched off, black). The x-axis represents the experimental input, and the y-axis represents the input node. The right side of the figure shows the seven sets of Ae models. The x-axis represents the source node, and the y-axis represents the target node. **(B)** The figure displays the correlation matrices corresponding to the “Bottom-up” sub-set of models from the first family-level inference. The left side of the figure shows the matrix C driving input, with the “Striatal node” as the parameter to be estimated, indicated in light blue, while the rest of the nodes are fixed at zero (switched off, black). The x-axis represents the experimental input, and the y-axis represents the input node. The right side of the figure shows the seven sets of Ae models. The x-axis represents the source node, and the y-axis represents the target node.

**Figure 5:**
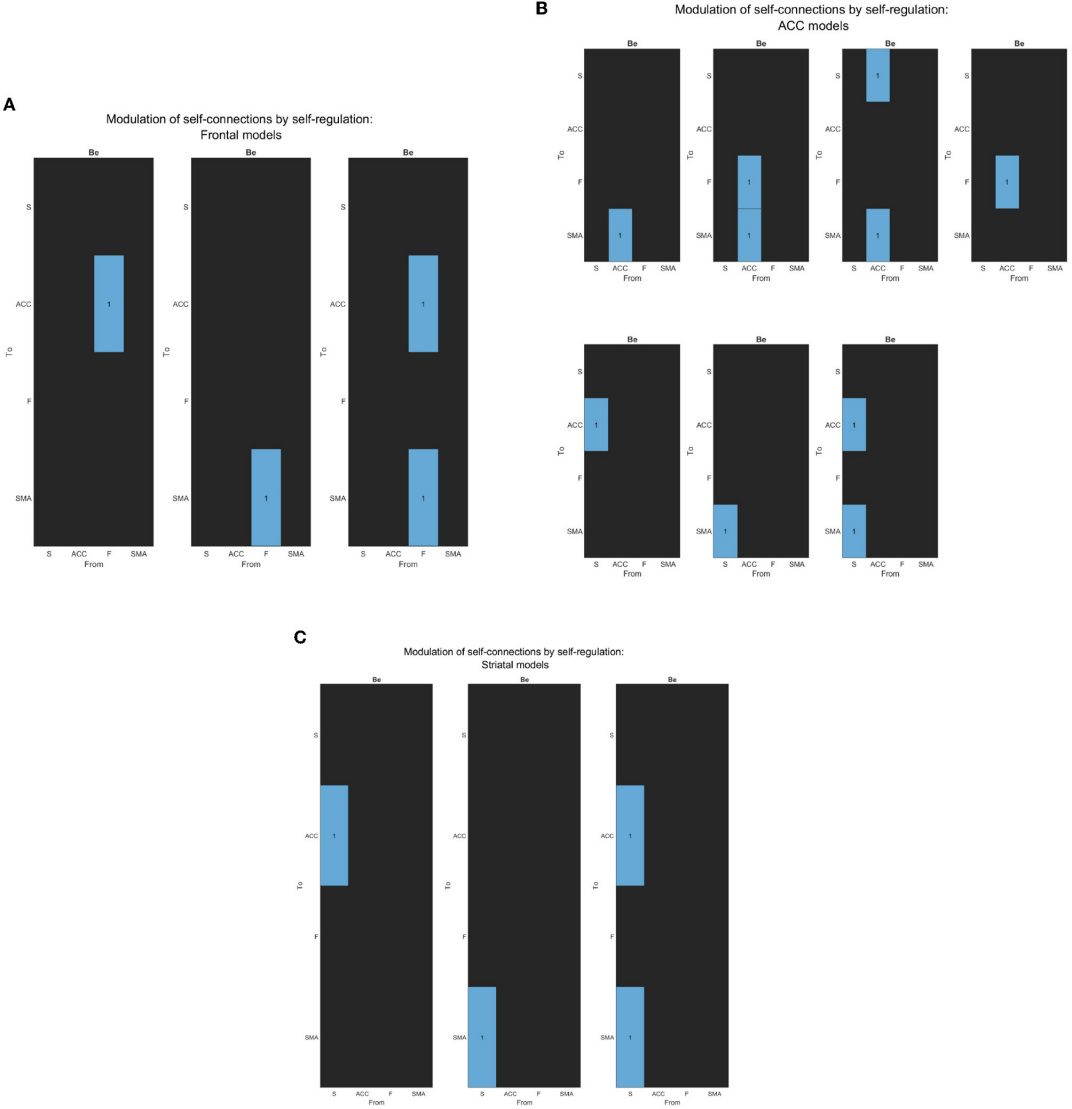
**(A)** The figure displays the correlation matrices corresponding to the “Frontal” sub-set of models from the second family-level inference. The figure shows three Be matrices, each of which has the “Frontal node” as the source node for modulation to other nodes. The parameter to be estimated is indicated in light blue, while the rest of the nodes are fixed at zero (switched off, black).The x-axis represents the source node, and the y-axis represents the target node. **(B)** The figure displays the correlation matrices corresponding to the “ACC” sub-set of models from the second family-level inference. The figure shows five Be matrices, each of which has the “ACC node” as the source node for modulation to other nodes. The parameter to be estimated is indicated in light blue, while the rest of the nodes are fixed at zero (switched off, black).The x-axis represents the source node, and the y-axis represents the target node. **(C)** The figure displays the correlation matrices corresponding to the “Striatal” sub-set of models from the second family-level inference. The figure shows three Be matrices, each of which has the “Striatum node” as the source node for modulation to other nodes. The parameter to be estimated is indicated in light blue, while the rest of the nodes are fixed at zero (switched off, black). The x-axis represents the source node, and the y-axis represents the target node.

To investigate which general model structure underlay successful brain up-regulation, we categorized the models into two types: Active inference (Friston et al., 2016; Pezzulo et al., 2018) and RL (bottom-up) (Huang et al., 2020). Based on previously identified brain areas in the selected study (Sepulveda et al., 2016), we aimed to identify a network consisting of F, ACC, and S cortices, which are known to play a role in self-regulation (Emmert et al., 2016; Sitaram et al., 2017; Watanabe et al., 2017; Cortese et al., 2020) and are supported by RL studies (Huang et al., 2020) and Active inference anatomy (Pezzulo et al., 2015). For each subject, the models estimated each training run. The first family-level inference procedures involved testing fourteen (14) models, including seven (7) baseline effective connectivity architecture models (matrix Ae) with different driving inputs (matrix C) (Figures 4B, 5A), defining the partition of the model space. Regarding the Ae, we focused on the Frontal-Anterior cingulate-striatum axis (F-ACC-S axis) configuration, which has been proposed in the Active inference (Pezzulo et al., 2015), RL (Huang et al., 2020), and self-regulation (Emmert et al., 2016; Sitaram et al., 2017; Watanabe et al., 2017; Cortese et al., 2020) literature. The Ae models varied in the connection along the axis and with the target areas, and we also included an additional Ae model with a direct S-F connectivity configuration to test the hypothesis that a relay area (ACC) is required. To explore the effects of driving inputs (matrix C) on the models, we partitioned the model space into two subsets. The first subset included the Active inference models with the input on the frontal region (Figure 4A), while the second subset included the models with the bottom input on the striatal region (Figure 4B). Using the optimal model from the first step, we build the second family-level inference procedures (Figure 3). This model space comprised a total of thirteen (13) models of modulation of effective connectivity (matrix Be). In order to identify the learning-related changes in the modulation of effective connectivity, we test our main research question: whether the effect of self-regulation was manifest in the connection from higher levels—i.e., from F nodes (Figure 5A)—or lower levels via the ACC (Figure 5B) or from striatal nodes (Figure 5C).

#### 2.3.2. Group comparison: parameter-level inference

We use Bayesian model average (BMA) within the second family-level inference to produce a representative summary of the most likely connectivity architecture and the effect of self-regulation. In order to compare parametric differences at the group level, a two-sample *t*-test (*p* < 0.05) was applied to the modulatory effects of self-regulation. We report Bonferroni corrected significant differences, comparing learners and non-learners.

### 2.4. Learning ranking

To ensure methodological rigor and prevent double-dipping, we employed the Wasserstein distance (WD) (Panaretos and Zemel, 2019) as a measure of learning in line with recent research (Yan et al., 2019). This distance metric allowed us to quantify the relationship between target area activity and cognitive performance by comparing the baseline run and the transfer run (Fede et al., 2020). Specifically, we computed the sample mean of the SMA BOLD activity for each subject's initial baseline and the final post-training trial. Using a Gaussian kernel, we estimated the probability density distributions of these two trials, and then calculated the WD between the two resulting one-dimensional distributions. The WD analysis was conducted using a Python implementation (Virtanen et al., 2020).

## 3. Results

In order to report how the best model was selected, we present the evidence obtained for the baseline effective connectivity architecture (matrix Ae) and the region that received the driving input of feedback (matrix C). We then report the modulation of effective connectivity by the effects of self-regulation (matrix Be). Finally, we present the parameter-level inference differences between groups and learning ranking results.

### 3.1. Family-level inference: within-subject analysis

**Figure 6:**
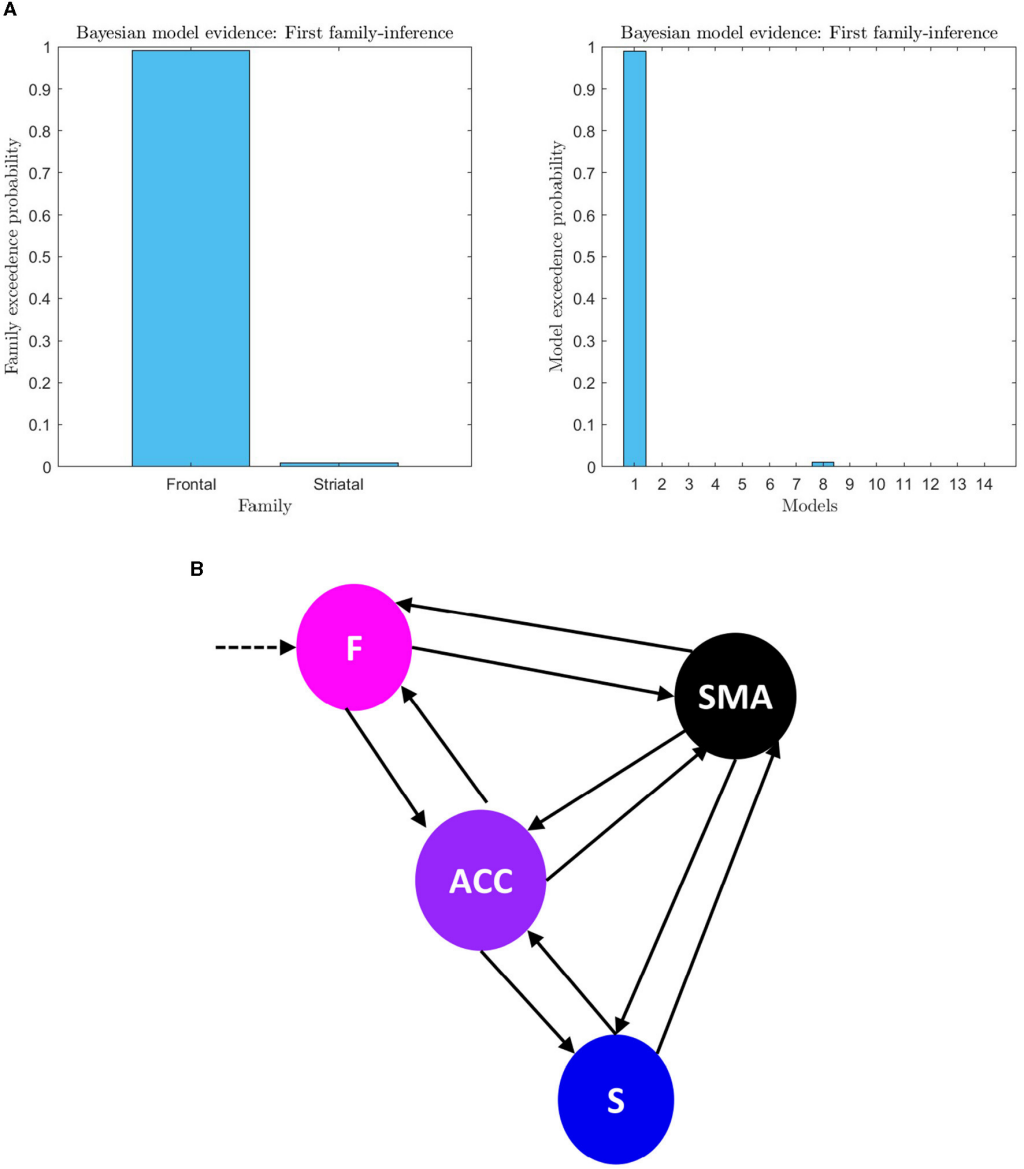
**(A)** First family-level inference. The figure shows the results of the first family-level inference. On the left side, the plot displays the family exceedance probability for the two families at the first family-level inference. The y-axis represents the probability, ranging from 0 to 1, and the x-axis represents the family indices, with family “Frontal” on the left and family “Striatal” on the right. On the right side, the plot shows the exceedance probability for the fourteen models. The probability values are presented on the y-axis, ranging from 0 to 1, and the x-axis shows the model indices. The “top-down models” are numbered 1–7, and the “bottom-up” models are numbered 8–14. The plot demonstrates the evidence of the first family-level inference at the family and model level by showing the exceedance probability (pxp). **(B)** First family-level winning model architecture. The architecture of the winning model at the family level is depicted, which includes bilateral effective connectivity in the F-ACC-S axis and bilateral connectivity from brain nodes to the target area. This model was identified as the winner through Bayesian Model Selection (BMS) with a high probability of 0.98 (pxp ≈ 1). Additionally, the family with the frontal lobe (F) driving input was identified as the winning model in all trials for all subjects with the probability of 0.99.

**Figure 7:**
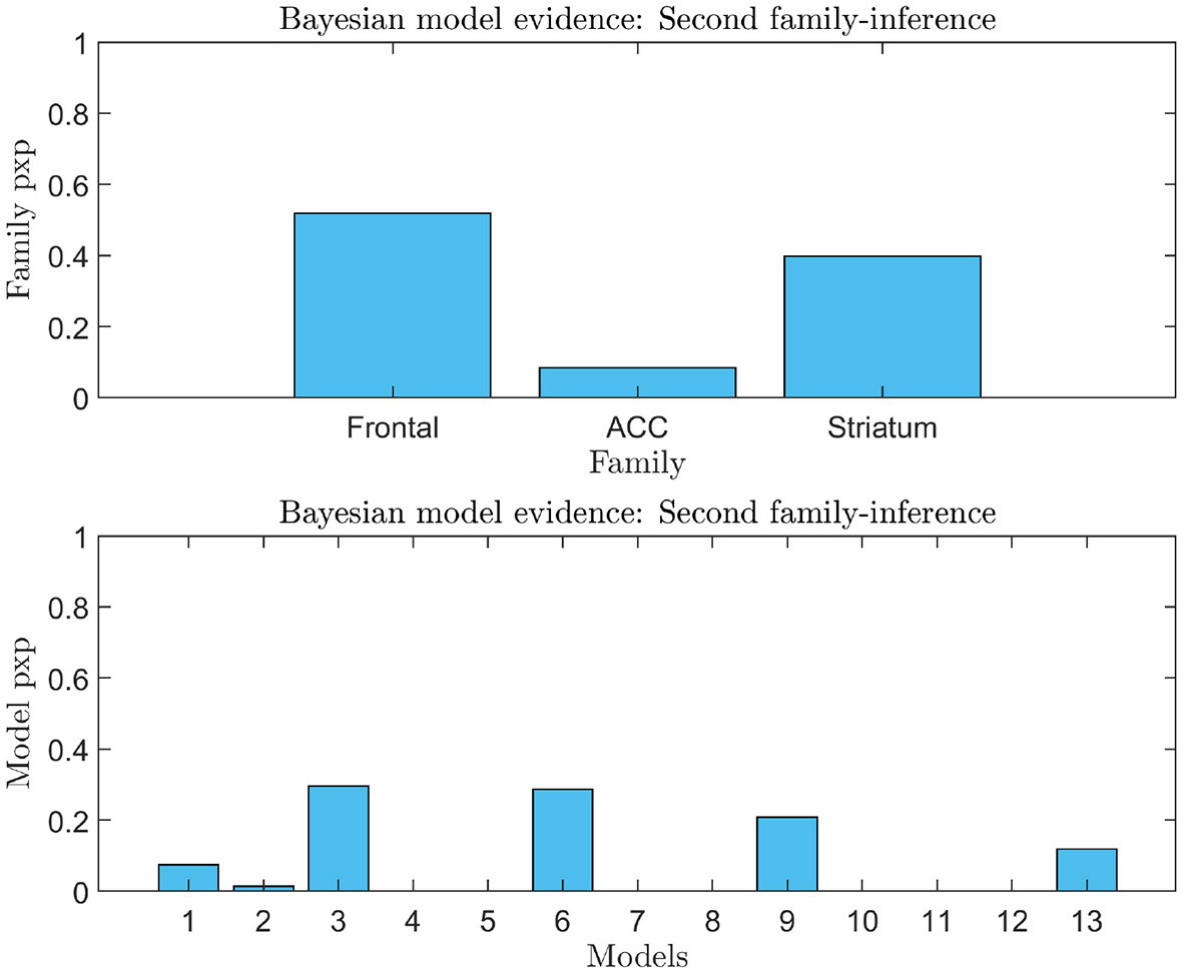
Second family-level inference. The figure shows the results of the second family-level inference. On the top the plot displays the family exceedance probability for the three families at the second family-level inference. The y-axis represents the probability, ranging from 0 to 1. The x-axis represents the family indices, with family “Frontal” on the left, family “ACC” in the center, and family “Striatal” on the right. On the bottom the plot displays the results of the second family-level inference by showing the exceedance probability (pxp) for the thirteen models considered. The probability values are presented on the y-axis, ranging from 0 to 1. The x-axis shows the model indices, with the “Frontal” models numbered 1–3, the “ACC” models numbered 4–8, and the “Striatal” models numbered 9–13.

An analysis of the evidence reveals that the optimal baseline effective connectivity architecture (matrix Ae) is the one with bilateral links across the F-ACC-S axis (Figure 6A) and the region that received the driving input of feedback (matrix C) is the Frontal node as the most likely (pxp ≈ 1; Figure 6A) (Figure 6B for a graphical description of the model). However, in the second family-level inference, which concerns the modulatory effect of self-regulation, any family and model exhibit a posterior model density with a pronounced peak. In the thirteen DCM, there is a pxp ≈0.5 at the level of the best family (Figure 7), and the best models of each family have a limited range of evidence (pxp [0.0005–0.2952]; Figure 7); hence, there is no clear dominant model identified. In order to perform a comprehensive identification of the self-regulatory network, we include the set of 13 DCMs and three families in the between-group analysis (Section 3.2).

### 3.2. Parameter-level inference: between-group analysis

**Figure 8:**
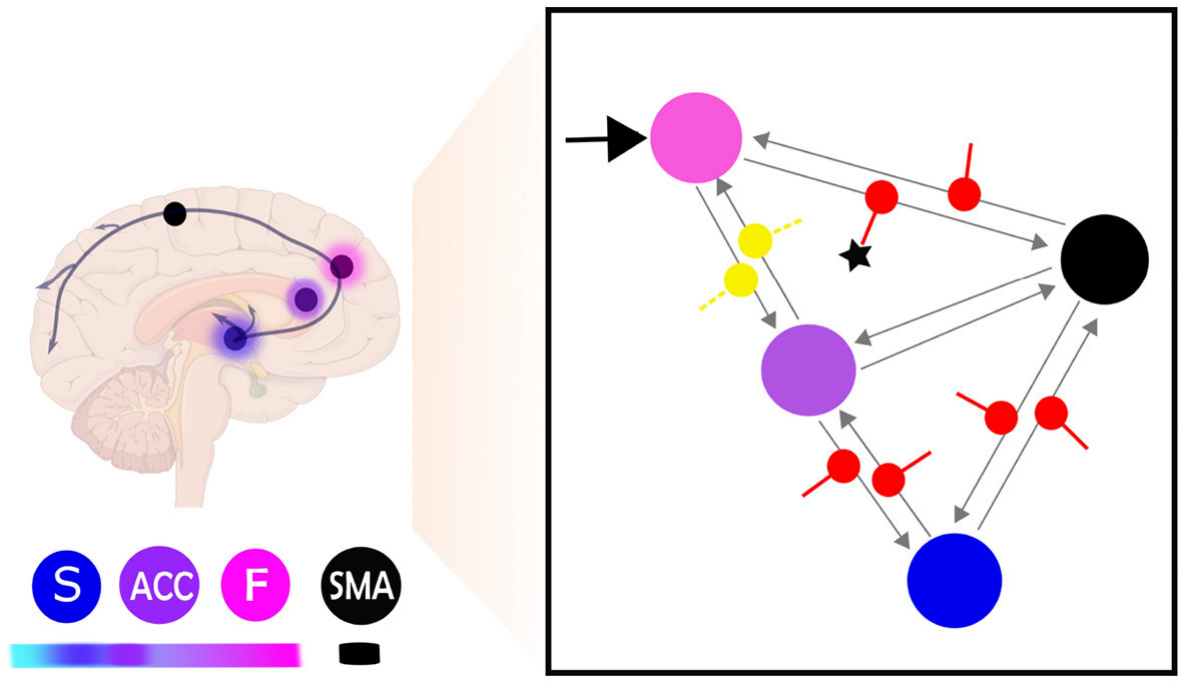
This figure provides a schematic illustration of the most probable model and parameter configuration of brain self-regulation learning. The right side of the figure shows the network connectivity during self-regulation and the significant difference between learners and non-learners. The input connection (C matrix) is indicated by a black arrow, while the effective connectivity architecture (Ae matrix) is represented by gray arrows. The modulation of extrinsic connectivity values (matrix Be) is depicted as red for excitatory and yellow for inhibitory. The star (⋆) indicates the modulatory connectivity that exhibits a significant difference (*p* < 0.05) between learners and non-learners. The left side of the figure shows the same network represented as brain regions, with nodes indicating the different brain regions and their hierarchical identification shown as color labels. Higher hierarchical levels are represented by darker fuchsia pink, while lower hierarchical levels are indicated by lighter blue (Pezzulo et al., 2015). The frontal lobe is labeled as F, the anterior cingulate cortex as ACC, the striatum as S, and the supplementary motor area as SMA.

Having identified the best model up until the matrix Ae and matrix C, we subsequently analyzed the model parameters resulting from the BMA within a fully modulatory effect model (matrix Be). In terms of group difference, averaged posterior estimate comparison indicated that self-regulation effects on F-SMA target connectivity in learners were significantly higher than in non-learner. Figure 8 reports the results of mean modulatory connection changes during self-regulation and the significant difference between groups.

### 3.3. Learning: difference in self-regulation performance

**Figure 9:**
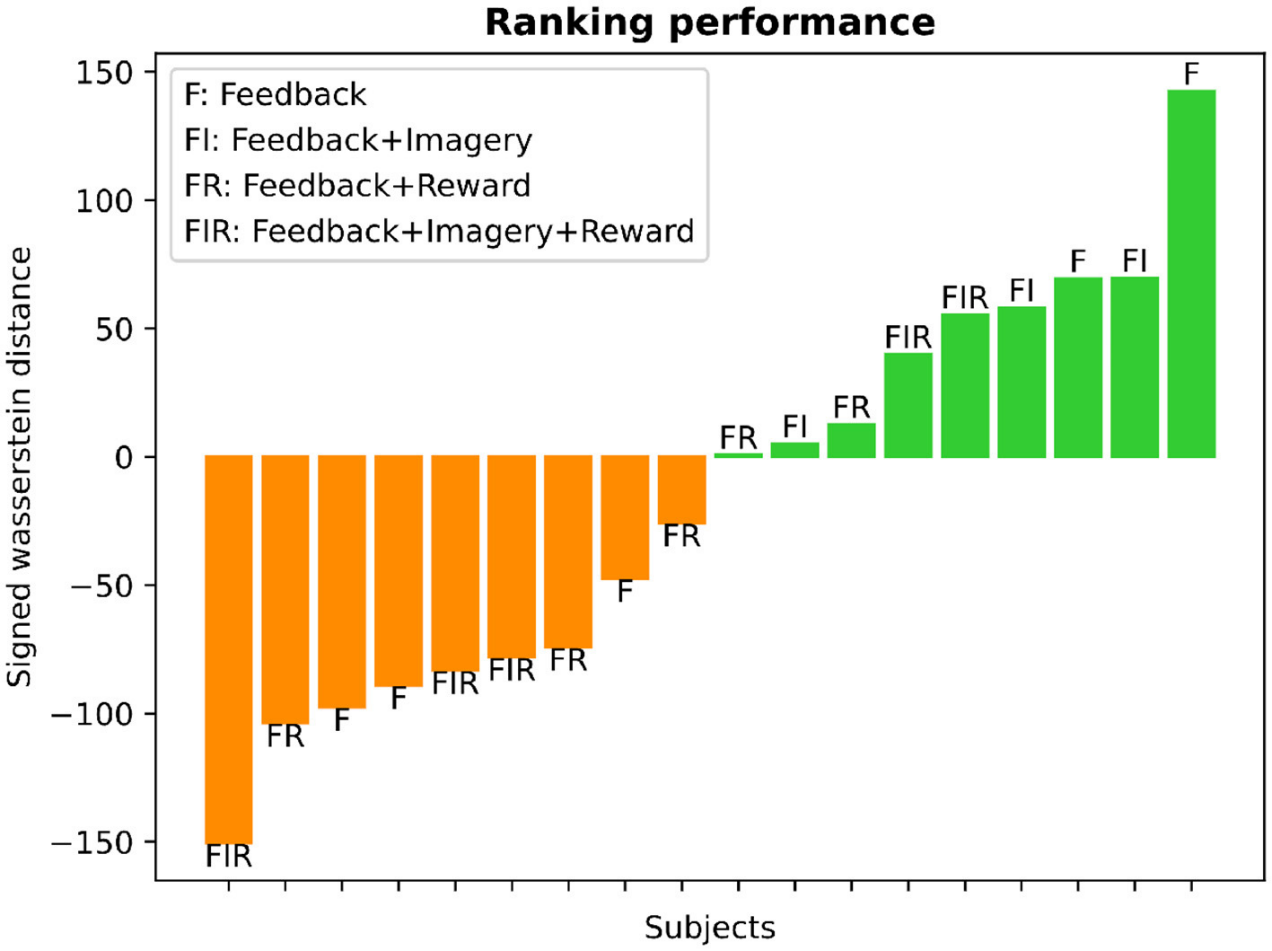
Learning ranking analysis. The histogram presents the SMA-BOLD performance for each subject, representing the change in performance (positive values for increase, negative values for decrease) measured using the Wasserstein distance. The distribution illustrates an equal split of 50% between learners and non-learners. The bar plot displays the subjects' performance categorized into different training groups, as defined by Sepulveda et al. (2016). Each bar is labeled at its edge to indicate the specific type of training: F (feedback), F+I (feedback and motor imagery), F+I+R (feedback, motor imagery, and reward), and F+R (feedback and reward).

The learning ranking analysis for each subject is depicted in Figure 9. We employed the Wasserstein distance (WD) between the mean BOLD-SMA activity during baseline and post-training to assess learning. The distribution of WD values revealed a balanced split, with approximately 50% of participants classified as learners and the remaining 50% as non-learners. In Figure 9, larger positive WD values are indicative of significant modifications in brain activity, reflecting successful up-regulation in response to the task goal. These instances are represented by the green color bars. Conversely, greater negative WD values suggest reduced activation of brain activity in relation to the training objective, representing down-regulation. These instances are visualized by the orange color bars.

## 4. Discussion

In this study, we used DCM to analyze a human real-time neurofeedback fMRI dataset by testing hypotheses about effective connectivity differences between neurofeedback learners and non-learners. To identify these differences, we compared neuronal architectures implied by RL with Active inference, where the brain learns an internal representation of the causes of the sensation, i.e., the sensory feedback signal that derives from its own activity. The self-regulation learning task was best modeled by a hierarchical (i.e., Active Inference) architecture, with the effects of feedback and self-regulation mediated by top-down connections from the F-ACC-S axis and the SMA. This contrasts with the alternative (i.e., RL) architecture, with the striatum as the central hub. The findings indicate different network modulation weights between neurofeedback learners and non-learners. Neurofeedback is increasingly being used as a tool for self-regulating brain activity and connectivity for neuropsychological rehabilitation (Sitaram et al., 2017). Little is known about the mechanisms of learning to self-regulate or why learning rates are relatively low, with “non-responder effect” quantified in rt-fMRI neurofeedback from 25% up to 75% of neurofeedback participants unable to learn the task (Fede et al., 2020). On the other hand, without prior information, the chance of encountering the causal link between some internal brain state and the sensory feedback presented to the participant is very low, given the size of the search space (Watanabe et al., 2017). The fact that at least 25% of participants succeeded in the task in the relatively short duration of the experiment should be seen to be well above the odds. The question becomes what strategy those participants apply to identify and then control the target brain state. The optimal design of self-regulation protocols also remains unclear. By understanding the characteristic changes in effective connectivity in learners and non-learners, future implementation of self-regulation can be optimized while finessing theoretical accounts of cognitive action and control. Our analysis shows that the self-regulation learning task of Sepulveda et al. (2016) was best modeled by a hierarchical (i.e., Active Inference) architecture, with the effects of feedback and self-regulation mediated by top-down connections from the F-ACC-S axis and the SMA—the target area for self-regulation learning. This contrasts with the alternative (i.e., RL) architecture, with the striatum as the central hub. The winning model structure calls for a reinterpretation of learning and neurofeedback studies, where it has been suggested that reward-related processes account for the neurofeedback changes in the pattern of brain activations (Sepulveda et al., 2016; Sitaram et al., 2017). Indeed, Active inference offers a reinterpretation (Pezzulo et al., 2015; Friston et al., 2017), where a shift from striatal bottom-up to Frontal top-down processing has been proposed as a better explanation of learning (Smith et al., 2020). It is worth noting that Active inference and RL models are not mutually exclusive. Active inference considers learning not only at the trial-error-reward (Mnih et al., 2015) level of learning but also in relation to configurations of hierarchical prior structures of the internal model (Parr et al., 2019; Smith et al., 2020). Consequently, the generative model is updated by bottom-up signals, increasing predictive validity of internal (i.e., generative) models. This enables better predictions and the resolution of (e.g., striatal) prediction errors. In other words, top-down predictions underwrite optimal inference and learning. In particular, it is the difference between top-down predictions in the form of Frontal prior beliefs—coupled with an initial stage of unreported actions—that appear to differentiate learners and non-learners. We argue that relying only on bottom-up signals during self-regulatory learning would be an untenable learning strategy due to the dimensionality of the search space and time constraints. For self-regulation learning to succeed, top-down control is mandatory. The neural substrate for the dynamics of the internal generative model (Pezzulo et al., 2015) is consistent with the network involved in learning (Huang et al., 2020), in which the F-ACC-S axis is present; also observed in previous studies in abstract construction (Collins and Frank, 2013) and cognitive control (Bassett et al., 2015). Previous studies have demonstrated the role of the frontal cortex as an encoder of new hidden states during inference (Cortese et al., 2020) and modulator of learning in its connections with lower areas (Bassett et al., 2015), such as primary motor cortex and supplementary motor cortex. It is important to acknowledge the limitations of the current study. Firstly, here we identified the learner and non-learner based on distance metric over the level in mean BOLD-SMA ROI values between baseline and post-training. This way of separating the subgroups may not be the only one, nor is it the most optimal, as the literature has suggested using sustained transfer effects as a method (Fede et al., 2020). We also acknowledge that pre-post training tests can be influenced by confounding factors such as fatigue, motivation, and habituation. It is essential for future protocols to address and track potential confounders such as habituation and motivation, such as analyzing habituation patterns (Gruzelier, 2014), as well as incorporating surveys to assess attention, motivation, and fatigue levels (Diaz-Garcia et al., 2021). By monitoring these variables, a more comprehensive understanding of the factors influencing learning outcomes can be obtained, thus enhancing the validity and reliability of the results. The cognitive strategies that participants used to approach the task vary considerably (Sepulveda et al., 2016). Here, we reported the results at the group level: i.e., learners vs. non-learners. Ideally, one would want to identify the individual models that participants use. However, the design of the original study and the sample size do not permit this level of analysis. Secondly, it is known that there is also variability in cognitive strategy (The cognitive strategies during training as described in Sepulveda et al., 2016), but due to data design, we were unable to identify at the subject level. Our results can only be compared to the transfer results presented in Figure 2 of Sepulveda et al. (2016) paper, which demonstrates sustained activity in the groups that received feedback (F) and feedback plus imagery (F+I). It is important to note that this differs from the findings in Figure 1, which illustrate the dynamics of learning and show increased activity in the reward conditions. This contrast is intriguing from the perspective of active inference, as it suggests that while rewards may enhance activity, they may not be sustainable for effective control, as proposed by the active inference hierarchical model. Based on the main results demonstrating top-down modulation of prefrontal cortical (PFC) regions on the target area, it is reasonable to consider that the presence of anatomical connections between the PFC and the target region may indicate a greater potential for self-regulation in the target area. However, it is important to note that while anatomical connectivity is a necessary condition, it alone does not guarantee successful self-regulation. Other factors related to the anatomical connections, such as the directness or indirectness of the connections, the strength of the connections (e.g., the number of fibers), and the presence of redundant connections, may also be important anatomical considerations. In addition to anatomical connections, other factors related to the functional and behavioral relevance of the target region to the individual could play a significant role in self-regulation. For example, specific cortical regions may be more amenable to self-regulation in individuals with expertise or experience in related domains. For instance, auditory cortical regions may be more easily modulated by singers, somatosensory areas by instrumentalists, and gustatory areas by cooks. Similarly, motor cortical regions may be more responsive to self-regulation in athletes. Conversely, certain populations, such as stroke patients, individuals with autism or schizophrenia, or those with tinnitus, may encounter challenges in regulating motor cortical regions, emotional brain regions, or auditory cortical regions, respectively. It is important to note that many of these hypotheses are yet to be tested and validated. Further research is needed to explore the complex interplay between anatomical connections, functional relevance, and self-regulation ability in various contexts and populations. From our results, it could be suggested that techniques that increase top-down connectivity that will enhance NF effectiveness. Further, studies should test the prediction by experimental manipulation of the frontal regions, i.e., Transcranial Direct Current Stimulation (tDCS), and by agent-environment simulations of prior configurations. We recommend that follow-up studies include more training trials and subjects for that allow greater statistical power for personalized analyses. Further studies could include behavioral tests and self-reports to evaluate task generalization relating to target region functionality to quantify the effectiveness of the NF. Finally, as with all dynamic causal modeling studies, we can only generalize our conclusions within the space of the models considered.

## 5. Conclusion

The results of this study provide new insights into the neural mechanisms underlying self-regulation learning. Specifically, we confirm the Active Inference model's prediction that the brain engages in top-down connectivity during learning rather than a bottom-up fashion as predicted by RL. In addition, we provide evidence that brain self-regulation is gated by the F-ACC-S axis; a pathway implicated in cognitive action control. Our findings also highlight the importance of the frontal-SMA target region connection on the effects of self-regulation. Furthermore, the present study showcases the Active Inference approach—coupled with modeling using the DCM—for testing hypotheses in NF-training fMRI at the group level. Here we extended the framework of AI to brain self-regulation learning, where self-regulation is a form of cognitive action similar to conscious access models, in which the neural target responses are controlled by one's own brain activity (Pezzulo et al., 2015; Chen et al., 2020). Taken together, the results deepen our knowledge about the mechanism of action of self-regulation learning. This may be particularly relevant for experimental setups and the development of new clinical therapeutics, which in turn, contribute to expanding our understanding of the mechanisms that underwrite cognitive action tasks.

## Data availability statement

Publicly available datasets were analyzed in this study. This data can be found here: https://github.com/galadriana/selfregulationlearning_DCM_fMRI.

## Ethics statement

The studies involving human participants were reviewed and approved by committee of Pontificia Universidad Católica de Chile. The patients/participants provided their written informed consent to participate in this study.

## Author contributions

GV performed the statistical analysis and visualizations and prepared the original draft. DA contributed to the methodology. PS contribute with the resources. MR-F supervised and contributed to manuscript editing. KF contributed to the conception and manuscript editing. RS contributed to the conception and supervised the manuscript editing. WE-D contributed to the conception, supervision, and manuscript editing. All authors contributed to the manuscript revision, read, and approved the submitted version.
