# Supplementary figures and images for: Self-regulation learning as active inference: dynamic causal modeling of an fMRI neurofeedback task
Source: Front Neurosci. 2023 Aug 15;17:1212549. doi: 10.3389/fnins.2023.1212549 (PMC10465165; doi:10.3389/fnins.2023.1212549)

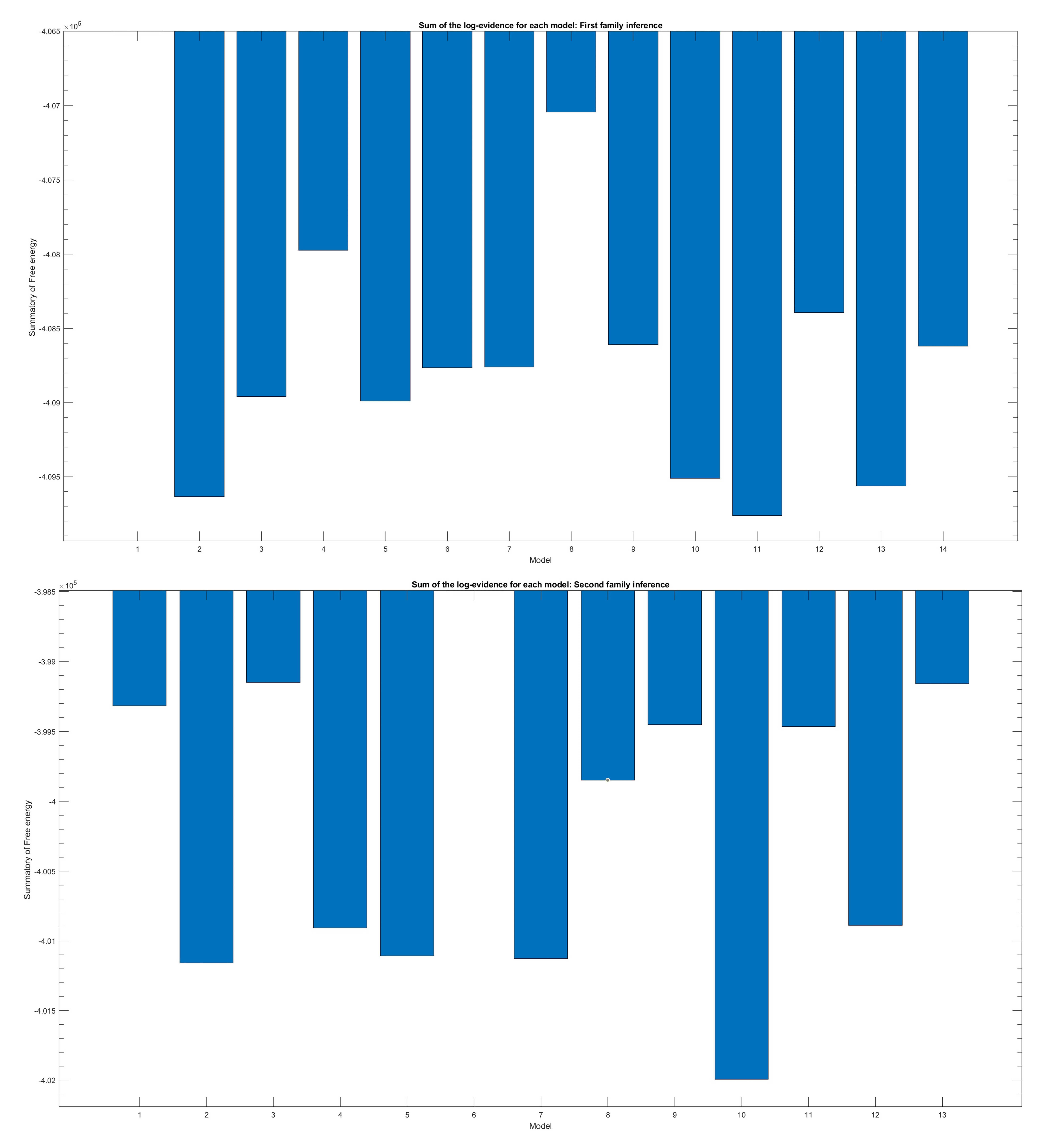

Supplement: Supplementary file 3 [file Image_1.JPEG]
